# Serum ferritin and incident cardiometabolic diseases in Scottish adults

**DOI:** 10.1186/s12933-022-01450-7

**Published:** 2022-02-16

**Authors:** Milton-Fabian Suárez-Ortegón, Stela McLachlan, José-Manuel Fernandez-Real, Tomi-Pekka Tuomainen, Alex Aregbesola, Sarah H. Wild

**Affiliations:** 1 Departamento de Alimentación Y Nutrición, Facultad de Ciencias de La Salud, Pontificia Universidad Javeriana Seccional Cali, Calle 18 No. 118-250, Cali, Colombia; 2 Grupo de Investigación en Ciencias Básicas Y Clínicas de La Salud, Facultad de Ciencias de la Salud, Pontificia Universidad Javeriana Seccional Cali, Cali, Colombia; 3 Usher Institute, University of Edinburgh, Edinburgh, UK; 4 Department of Diabetes, Endocrinology and Nutrition, Institut d’Investigació Biomèdica de Girona (IdIBGi), CIBERobn Fisiopatología de La Obesidad Y Nutrición (CB06/03/010) and Instituto de Salud Carlos III (ISCIII), and Department of Medical Sciences, School of Medicine, University of Girona, Girona, Spain; 5 Institute of Public Health and Clinical Nutrition, University of Eastern Finland, Kuopio, Finland; 6 Present Address: Department of Paediatrics and Child Health, Max Rady College of Medicine, Rady Faculty of Health Sciences, University of Manitoba, Winnipeg, MB Canada; 7 Children’s Hospital Research Institute of Manitoba, Winnipeg, Canada

**Keywords:** Metabolic syndrome, Iron metabolism, Obesity, Type 2 diabetes, Cardiovascular disease, Cerebrovascular disease

## Abstract

**Background:**

Iron stores, estimated as ferritin levels, and type 2 diabetes (T2D) have been associated previously, while findings regarding coronary heart disease (CHD) and cerebrovascular disease (CEVD) are still inconclusive. No study has focused on simultaneous evaluation of associations between iron stores and the above cardiometabolic diseases (CMD) in the same population. We aim to evaluate the association between serum ferritin and risk of T2D, CHD and CEVD in Scottish population over a wide range of ferritin levels.

**Methods:**

Longitudinal study in 6,497 participants of the 1995 and 1998 Scottish health surveys, who were followed-up until 2011. Cox regression models were conducted adjusting for age, sex/menopausal status, fibrinogen, GGT levels, smoking, alcohol consumption, total cholesterol, HDL-cholesterol, blood pressure, and BMI. Ferritin was used as continuous (sex/menopausal status-specific Z score) and categorical variable (sex/menopausal status-specific quartiles, quintiles and sextiles).

**Results:**

During follow-up, 4.9% of the participants developed T2D, 5.3% CHD, and 2.3% CEVD. By using ferritin quartiles, serum ferritin was positively associated with T2D, CHD and CEVD but only the association with T2D remained after adjustment for covariates [Quartile 4 v. 1: adjusted HR 95% CI 1.59 (1.10–2.34); P = 0.006]. When ferritin sextiles were used (6 v. 1), the ferritin-CEVD association became slightly stronger and significant [adjusted HR 95% CI 2.08 (1.09–3.94); P = 0.024].

**Conclusions:**

Iron stores relate differently to each CMD. Serum ferritin levels were positively and independently associated with incident T2D, and with incident CEVD if higher cut-off points for high ferritin levels were considered.

**Supplementary Information:**

The online version contains supplementary material available at 10.1186/s12933-022-01450-7.

## Background

Increased iron stores, reflected by high serum ferritin levels, have been associated with the development of type 2 diabetes (T2D) [1]. Reports on the association between iron stores and other cardiometabolic diseases (CMDs) (coronary heart disease (CHD), cerebrovascular disease (CEVD)) for which diabetes is also a risk factor are inconsistent [2]. Few studies have investigated the association between iron metabolism and CEVD. To date, no published study has focused on simultaneous evaluation of associations between iron stores and CMDs in the same population or in nationally representative samples. In addition, there has been limited exploration of potential threshold effects of ferritin concentration on the risk for CMDs. Although the shape of serum ferritin-T2D risk have been studied in a few studies, the shape of relationships between serum ferritin and CHD or CEVD have not been investigated.

In Scotland, CHD persists as a leading cause of illness and death [3, 4] and prevalence of all types of diabetes has increased over the last decade, from 3.2 to 5.1%. Increasing prevalence of diabetes is partially explained by decreasing T2D mortality and stable or small declines in T2D incidence [5]. However, there is no information on iron biomarkers as a potential novel risk factor for CMD in the Scottish population.

The description of the relationship of serum ferritin with all types of CMDs in a population at high cardiovascular risk would provide better understanding of the link between iron metabolism and overall cardiometabolic risk. As the distribution of iron biomarkers, confounders, covariates and outcomes can be highly variating among populations from different countries and regions, comparability between studies become harder as well as overall interpretation of findings. Since diabetes and cardiovascular disease are CMD, using a same population represents a context in which both evaluations, iron-diabetes and iron-cardiovascular disease, are conducted under the same analytical conditions providing an association pattern with regard CMD rather than for a single disease event separately. Hence, we investigated the risk of T2D, CHD, and CEVD in participants in the 1995 and 1998 Scottish health surveys (SHeS), who were followed-up until 2011, the most recent point for which data linkage was feasible, using a wide range of serum ferritin levels.

## Methods

The SHeS 1995 and 1998 included participants aged 16–74 years. The surveys randomly selected a nationally representative, general population sample (see The Scottish Health Surveys websites for more detail) [6, 7]. All participants were interviewed about health and lifestyle behaviours, and consent for measurement of weight and height and collection of a blood sample was requested. The SHeS also has a prospective element on the basis of linkage to population level data on hospitalization and mortality. This linkage is facilitated by the Information Service Division (ISD) which collects and updates data on deaths from the General Register Office and morbidity derived from hospital discharge data in Scottish Morbidity Records (SMR) and provides linkage to SHeS data [8]. There is also a retrospective element with data—linked to morbidity since 1981. Around 90% of SHeS participants provided consent for data linkage [8]. As a consequence of the wording of the consent for data linkage only data from the 1995 and 1998 SHeS have been linked to diabetes register data.

There were 9568 participants whose ferritin levels were measured and who agreed to have their data linked to the Scottish Morbidity Record (SMR), the Scottish Diabetes Register, and death records. Among these, we excluded cases of prevalent T2D, CHD, and CEVD at baseline (defined as interview day), subjects who had type 1 diabetes (T1D) at baseline or who developed T1D during follow-up, cases with missing values for covariates (listed in data analysis section), and those below the set age limit (age ≥ 18 years). Additional file 1: Fig. S1 describes the selection of the analytical sample (*n* = 6497). The secondary analysis of the SHeS-linked dataset was approved by the Ethics Research Subgroup of the Centre for Population Health Sciences of the University of Edinburgh.

### Biochemical and clinical variables

The exposure variable, serum ferritin, was measured by the Abbott Microparticle Enzyme Immunoassay (MEIA) /IMX ferritin assay method in 1998, and in 1995 it was used the Boehringer Enzyme Immunoassay (EIA) method instead. Both methods are non-radiometric and a recent study by Garcia-Casal et al. reported a highly comparability among different ferritin assays including radiometric methods in a systematic review and meta-analysis [9]. For non-radiometric assays such as MEIA and EIA, the correlation coefficient was 0.989.

### Methods for biochemical and clinical variables

Blood was collected via venepuncture but not after overnight fasting, given that the only biochemical cardiovascular risk factors to be measured were cholesterol markers. After food intake, these markers of lipid profile appear to be, at most, minimally changed and have shown good prediction of increased risk for cardiovascular disease [10]. Levels of HDL-C were measured by using enzymatic-colorimetric method. Fibrinogen level was estimated by nephelometric method (clot turbidity). Coulter analysers were used to measure haemoglobin, and the nitroanilide method was used for GGT.

Blood pressure was measured using mercury sphygmomanometers with an appropriately sized cuff in a sitting position after 15 min of rest. Phase I and V (disappearance) Korotkoff sounds were used to identify SBP and DBP [11]. Three blood pressure readings were taken and the average of the second and third readings was used for the analyses. Body weight and height were measured using standard techniques and instruments [12]. BMI was calculated as weight in kg/height in metres squared [13]. WC was measured from the midpoint between the lateral iliac crest and the lowest rib using a flexible steel tape measure [14]. The criterion of smoking included the following categories: never smoker, ex-regular or ex-occasional smoker, and current smoker. The criterion of alcohol intake included the following categories, using rating units per week: (1) non-drinker or ex-drinker, (2) trivial drinker < 1 rating unit per week, and (3) drinker ≥ 1 rating unit per week. Anaemia was defined as haemoglobin < 13 g/dL in men and < 12 g/dL in women [15]. Overweight and obesity were defined as having a BMI ≥ 25 and BMI ≥ 30, respectively. Physical activity levels, an additional variable used in this analysis, were estimated as a summary of self-reported work, walking, and sport activities in categories of intensity levels as inactive, light, moderate, and vigorous [16].

### Cardiovascular outcomes and T2D

Fatal and non-fatal cardiovascular events recorded in hospital admissions were identified during the follow-up period. Coronary heart disease (CHD) included angina, myocardial infarction, and other acute ischemic heart disease (defined using ICD-10 codes I20–I25, ICD-9 codes 410–414) [17]. CEVD included stroke, nontraumatic subarachnoid haemorrhage or intracerebral haemorrhage, other and unspecified nontraumatic intracranial haemorrhage, cerebral infarction, occlusion and stenosis of precerebral and cerebral arteries, and category of other cerebrovascular diseases and transient ischemic heart attack (defined using ICD-10 codes I60–I67, G-45; ICD-9 codes 430–437) [17]. Incident cases of T2D were identified from relevant codes ICD-10 and ICD-9 (E11–E14, 250) recorded in hospital admissions during follow-up and from the linked data derived from the population based register of diagnosed diabetes which is estimated to have been complete since 2004. Codes for unspecified diabetes in hospital records were as assumed to describe T2D. Records from which outcomes are identified are population based expecting all diagnosed diabetes and all hospital admissions for CHD.CEVD to be identified.

### Data analysis

Medians and their interquartile ranges and proportions were used for description of continuous and categorical study variables, respectively, in the whole sample and by sex-specific and self-reported menopausal status-specific quartiles of ferritin concentration. Trends of distribution of study variables across ferritin quartiles were tested by the Jonckheere-Terpstra test [18] (continuous variables) and χ^2^ test (categorical variables). For the analyses of association, ferritin levels were used as both continuous and categorical variables. For the continuous approach, we calculated a sex/menopausal status-specific Z score for ferritin, after log-normalisation of the ferritin values. The Z score enabled reporting of the risk for CMD by increasing SD units of log-ferritin. The categorical approach involved the use of sex/menopausal-specific quartiles of ferritin, with the lowest quartile as the reference category.

Cox regression models were used to examine the longitudinal associations between ferritin and CMD. HRs were described as unadjusted, age- and sex/menopausal status-adjusted, and fully adjusted for fibrinogen levels, GGT levels, smoking, alcohol consumption, total cholesterol, HDL-C, systolic blood pressure (SBP) and diastolic blood pressure (DBP), and BMI. This set of covariates was chosen on the basis of their possible influence in the ferritin-CMD associations. For instance, subclinical inflammation (fibrinogen), adiposity (BMI), liver injury (GGT), may affect circulating ferritin levels given that ferritin additionally could behave as acute phase reactant, injured hepatocytes can release ferritin into bloodstream, and fat mass or adiposity-related inflammation may increase ferritin concentration. Inflammation, liver dysfunction and adiposity in turn have also been extensively associated with CMD and thus they might behave as confounders. Meanwhile, adjustment for well-known cardiovascular risk factors (smoking, alcohol consumption, total cholesterol, HDL-C, SBP and DBP) helps to establish the influence of baseline cardiovascular risk of the individuals on the ferritin-CMD associations.

The proportional hazards assumption was tested using the Schoenfeld residuals test and graphical methods. The follow-up time was calculated from the date of survey interview (1995 or 1998) to the earliest date of incident T2D, cardiovascular event, death, or end of December 2011. Follow-up was censored at date of death as is appropriate for aetiological research [19]. Potential threshold effects of ferritin concentration were additionally investigated by comparing extreme quintiles and sextiles to extend the difference between extreme values beyond that offered by quartiles. To explore the shape of the relationship between serum ferritin and each incident CMD, we used restricted cubic splines. Since we explored use of different categories of ferritin concentration (sextiles in addition to quartiles) we used five knots as the maximum, with knots at the following percentiles of ferritin: 5th, 27.5th, 50th, 72.5th, and 95th, as suggested by Harrell [20].

Sensitivity analyses were performed on the basis of exclusion of subjects with clinically increased ferritin (> 200 µg/L in women and > 300 µg/L in men), potential liver disease (defined as GGT > 84 IU/L in men and > 44 IU/L in women), evidence of inflammation or infection (defined as fibrinogen levels > 4.7 g/L in 1995 and > 3.8 g/L in 1998) in sex/menopausal status-specific analyses.

Further adjustments for physical activity, self-reported hypertension, waist circumference (WC), and C reactive protein (CRP) levels as a systemic inflammatory marker, were also conducted. The adjustment for CRP was only performed for the participants from the SHeS 1998 in which this marker was measured. Before conducting the association analyses, continuous covariates with skewed distributions (fibrinogen, GGT, CRP, and WC) were log-transformed to approximate to normal distributions. Survey weights were applied to adjust for disproportionate sampling, differing selection probabilities, and differential non-response. All analyses were processed using STATA 14.0 software (Statistics/Data Analysis, Stata Corporation, 4905 Lakeway Drive, College Station, TX 77845, USA, 800-STATA-PC).

## Results

### Study variables by ferritin levels

The study variables by sex/menopausal status-specific ferritin quartiles are described in Table 1. Age, fibrinogen, GGT, BMI, total cholesterol, and blood pressure significantly increased across ferritin quartiles. The same pattern was observed for prevalence of current smokers and higher alcohol intake, and for the proportion of subjects who developed diabetes, CHD, and CEVD during follow-up. In contrast and as expected from the other risk factor patterns, HDL-C levels decreased with higher levels of ferritin. There was also a trend of a slightly increasing higher proportion of participants from SHeS 1995 vs. participants from 1998 throughout ferritin quartiles (Table 1). Ferritin levels were higher in men than women, and higher in post-menopausal women than pre-menopausal women (P < 0.05, data not shown).

**Table 1:** Baseline characteristics of participants and incidence of outcome diseases by sex-and menopausal stats-specific quartiles of ferritin level in the study cohort (n = 6497) [weighted values]

	All	Ferritin quartiles (µg/L)				
		Q1	Q2	Q3	Q4	P for trend
Age	41 (31–52)	39 (29–50)	40 (30–50)	40 (31–52)	44 (34–54)	< 0.001
Sex (Pre-W/Post-W/ Men),%)	32.9/16.6/50.5	32.9/16.4/50.7	32.2/16.9/50.9	33.3/17.2/49.5	33.0/15.9/51.1	0.887
BMI (Kg/mts^2^)	25.7 (23.1–28.6)	24.9 (22.7–27.8)	25.3 (22.8–28.1)	25.8 (23.3–28.5)	26.6 (24.1–29.9)	< 0.001
Systolic blood pressure (mmHg)	126 (117–137)	125 (117–135)	125 (116.5–135)	125.5 (116.5–137)	128.5 (118–140)	< 0.001
Diastolic blood pressure (mmHg)	70 (63–79)	69 (62–77)	69 (62–78)	70 (64–78)	73 (65–81)	< 0.001
Smoking status (%)						
Never smoker	39	45.4	41.1	36.1	33.0	< 0.001
Ex-regular or Ex-occasional smoker	26.3	24.4	26.3	26.1	28.4	
Current smoker	34.7	30.2	32.6	37.8	38.6	
*Alcohol consumption. Categories of rating units/week Prevalence (%)*						< 0.001
Never drank	4.2	5.1	5.4	3.6	2.6	
Ex-drinker	2.8	3.2	3.1	2.7	2.4	
Trivial drinker/Non-zero but under 1	10.3	12.6	10.7	9.1	8.5	
1–20	63.4	64.5	62.9	65.4	60.6	
≥ 21	19.3	14.6	17.8	19.2	26.0	
Total cholesterol (mmol/L)	5.4 (4.7–6.2)	5.3 (4.6–6.0)	5.4 (4.6–6.2)	5.4 (4.8–6.2)	5.6 (4.9–6.4)	< 0.001
HDL-cholesterol (mmol/L)	1.4 (1.2–1.7)	1.4 (1.2–1.7)	1.4 (1.2–1.7)	1.4 (1.2–1.7)	1.4 (1.1–1.7)	0.007**
GGT (IU/L)	20 (14–32)	17 (13–25)	18 (14–27)	21 (15–33)	27 (18–45)	< 0.001
Fibrinogen (g/L)	2.9 (2.4–3.5)	2.8 (2.3–3.4)	2.9 (2.4–3.4)	3.0 (2.5–3.6)	3.1 (2.6–3.7)	< 0.001
Ferritin (µg/L)	61 (32–108)	25 (13–43)	62 (28–78)	91 (42–116)	161 (86–216.8)	< 0.001
Ferritin range by sex/menopausal status*						
Premenopausal women (Pre-W) (*n* = *2239*)	2.0–950	2.0–18	19–30	31–47	48–950	
Postmenopausal women (Post-W) (*n* = *1343*)	3.0–1000	3.0–34	35–58	58–91	92–1000	
men (*n* = *2915*)	3.0–2251	3.0–61	62–96	97–151	152–2251	

Data for continuous variables are median (interquartile range) *Samples sizes, quartiles and ranges of ferritin levels are based on unweighted values

During a mean-follow-up of 14.1 (SD 2.8) years, 4.9% of the participants developed T2D, 5.3% developed CHD, and 2.3% developed CEVD.

### Association between serum ferritin and different CMD

Table 2 shows the HRs for the longitudinal association of serum ferritin levels, as Z score and quartiles, with the different CMDs. In unadjusted and age- and sex/menopausal status-adjusted models, ferritin as a continuous variable was positively and significantly associated with all types of incident CMD. The associations of ferritin as continuous variable with CEVD and CHD were no longer statistically significant after full adjustment for covariates. Similarly, for CHD and CEVD, the HRs for associations comparing highest to lowest quartiles of ferritin, were attenuated after adjustments when compared to unadjusted models and were no longer statistically significant (Table 2). (Table 2). In fully adjusted models, individuals with high levels (highest quartile) of ferritin had 1.70 times the risk of developing T2D as compared to people with low concentrations (lowest quartile) (P = 0.002), and a one SD increase in log ferritin was associated with higher risk of T2D [HR IC 95% 1.22 (1.08–1.38) P = 0.001] (Table 2).

**Table 2:** HRs and 95% CI for the incidence of diabetes and cardiovascular diseases by serum ferritin levels

	Type 2 diabetes			Coronary heart disease			Cerebrovascular disease		
	Unadjusted	Age- and sex/menopausal status- adjusted	Fully adjusted*	Unadjusted	Age- and sex/menopausal status- adjusted	Fully adjusted*	Unadjusted	**Age- and sex/menopausal status- adjusted**	Fully adjusted*
Z score of log-ferritin	**1.57 (1.39–1.78) ** **P < 0.001**	**1.45 (1.28–1.64) ** **P < 0.001**	**1.2 (1.07–1.39) ** **P = 0.002**	**1.22 (1.08–1.38) ** **P = 0.001**	**1.11 (1.00–1.25) ** **P = 0.049**	1.02 (0.90–1.15) P = 0.669	**1.28 (1.09–1.51)** ** P = 0.002**	**1.18 (1.01–1.37)** ** P = 0.029**	1.12 (0.95–1.33) P = 0.163
*Ferritin*									
Quartile 1	1.00 (reference)	1.00 (reference)	1.00 (reference)	1.00 (reference)	1.00 (reference)	1.00 (reference)	1.00 (reference)	1.00 (reference)	1.00 (reference)
Quartile 2	1.01 (0.67–1.50)	0.95 (0.64–1.41)	0.99 (0.66–1.50)	1.02 (0.71–1.46)	0.95 (0.66–1.36)	0.89 (0.61–1.29)	1.10 (0.66–1.82)	1.04 (0.62–1.73)	1.05 (0.63–1.75)
Quartile 3	1.34 (0.92–1.97)	1.23 (0.84–1.80)	1.10 (0.73–1.65)	1.04 (0.74–1.47)	0.93 (0.66–1.31)	0.79 (0.56–1.13)	1.23 (0.75–2.00)	1.11 (0.68–1.81)	1.05 (0.63–1.75)
Quartile 4	**2.73 (1.94–3.85)**	**2.28 (1.61–3.21)**	**1.59 (1.10–2.34)**	**1.70 (1.23–2.36)**	1.35 (0.97–1.87)	1.07 (0.76–1.51)	**1.86 (1.18–2.95)**	1.52 (0.96–2.40)	1.36 (0.81–2.27)
P for trend	**P < 0.001**	**P < 0.001**	**P = 0.006**	**P = 0.002**	P = 0.070	P = 0.700	**P = 0.007**	P = 0.065	P = 0.253

^*^ Adjusted for age, sex/menopausal status, fibrinogen levels, GGT levels, alcohol intake, smoking, systolic blood pressure, diastolic blood pressure, total cholesterol, HDL cholesterol, body mass index and year of survey

### Association between serum ferritin and the different CMD using additional categories of ferritin concentration

**Fig. 1:**
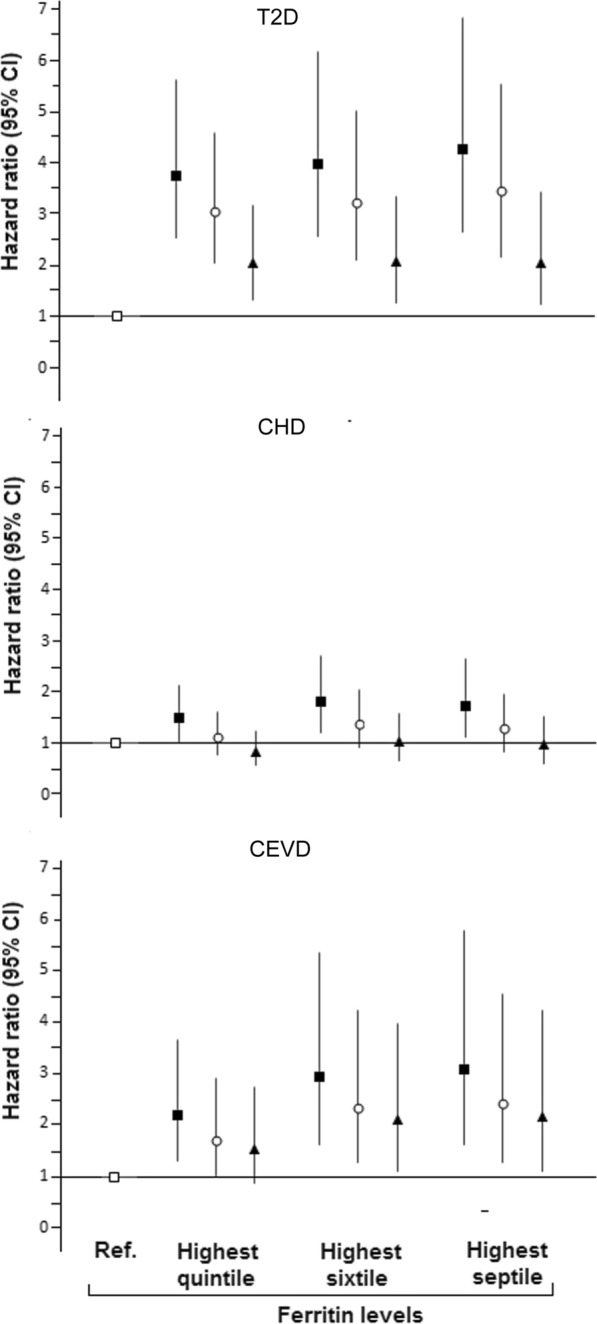
Risk of type 2 diabetes (T2D), coronary heart disease (CHD) and cerebrovascular disease (CEVD) by several sex-specific upper quantiles of ferritin levels (v. respective lowest categories). Ferritin range for highest quintile: Premenopausal women (Pre-MW) 53–950 µg/L, Postmenopausal women (Post-M) 102–1000 µg/L, Men 169–2251 µg/L. Ferritin range for highest sixtile: Pre-MW 58–950 µg/L, Post-MW 114–1000 µg/L, Men 183–2251 µg/L. Ferritin range for highest septile; Pre-MW 62–950 µg/L, Post-MW 124–1000 µg/L, Men 197–2251 µg/L.  Reference (lowest quintile, sixtile or heptile).  Unadjusted.  Adjusted for age and sex/menopausal status.  Adjusted for age, sex/menopausal status, fibrinogen levels, GGT levels, alcohol intake, smoking, systolic blood pressure, diastolic blood pressure, total cholesterol, HDL cholesterol, body mass index and year of survey. The above analysis included survey weights

When we explored the effect of using quintiles or sextiles to determine if the highest category of ferritin had a stronger association with CMD, there was a suggestion of an effect for CEVD, although confidence intervals for HRs overlapped (Fig. 1C). The hazard of CEVD was approximately double in individuals with ferritin in the highest sextile compared to those in the lowest sextile [fully adjusted (HR) 95% CI 2.08 (1.09–3.94), P = 0.024]. The association with T2D was slightly stronger when comparing extreme quintiles or sextiles than when comparing extreme quartiles (Fig. 1A), and no significant associations with CHD were observed (Fig. 1B). It is worth noting that the sex/menopausal status-specific cut-off points for the highest quintile and sextile were in the normal clinical range for serum ferritin (≤ 300 µg/L) (Fig. 1).

### Shape of the associations

**Fig. 2:**
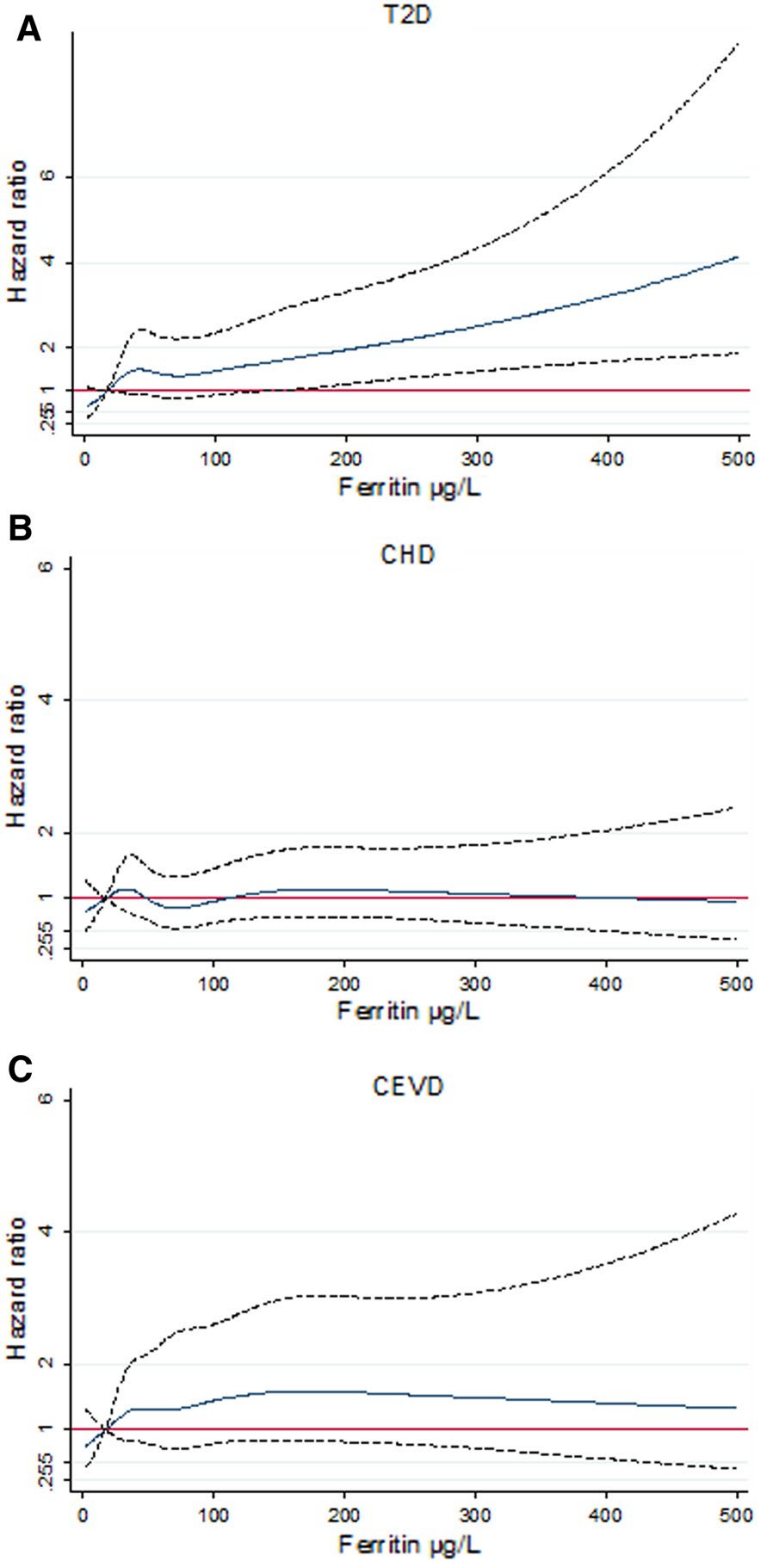
Risk of cardiometabolic diseases by serum ferritin levels. Adjusted hazard ratios for **A** T2D, **B** CHD, and **C** CEVD by serum ferritin levels .Ferritin levels higher than 500 µg/L were not used because confidence intervals tended to be very wide in this graphical analysis

The relationship between serum ferritin levels and incident T2D was approximately linear (Fig. 2A). In contrast with the findings by using sextiles of ferritin, the association between ferritin and CEVD was not observed when using cubic splines in terms of ferritin levels (Fig. 2C). This was perhaps because the ferritin values used for the evaluation of non-linear relationships were not sex/menopausal status-specific as they were in the analyses with sextiles of ferritin. When sex/menopausal status-specific Z scores of ferritin were used in the cubic splines analysis, a threshold effect of higher risk of CEVD appeared around + 1.20 SD of log-ferritin, although confidence intervals in that region of the graph still included 1 (Additional file 1: Fig. S2). The above findings on the shape of the association between ferritin or ferritin Z score and incident CEVD remained unmodified by using six knots instead of five in the cubic spline analysis. There was no evidence of an association linear or non-linear between ferritin levels and CHD in the cubic spline analysis (Fig. 2B).

We investigated the potential for a U-shaped association between ferritin and CMD. To test this, we used either ferritin quintiles as quartiles using 3rd quintile and quartiles 2–3 grouped as the reference categories. No associations were found between low ferritin (quartile 1 vs. quartiles 2–3 or quintile 1 vs. 3rd quintile) and CMD in adjusted models suggesting that a U-shaped association is not present (Additional file 1: Table S1).

### Analyses by sex

Although there was no evidence of interaction between sex or menopausal status and ferritin levels in respect to developing CMD (P > 0.05 in weighted and unweighted interaction tests), we conducted analyses stratified by sex and menopausal status. In these analyses (Additional file 1: Table S2) the statistically significant ferritin Z score-T2D association persisted only in men [HR (95% CI) 1.20 (1.01–1.43), P = 0.033; pre-menopausal 1.25 (0.92–1.69), P = 0.149; post- menopausal women 1.14 (0.87–1.49), P = 0.325]. When cases with ferritin levels above the normal range were excluded, the association between ferritin Z score and T2D was not significant in any of sex/menopausal status groups [pre- menopausal women HR (95% CI) 1.32 (0.96–1.82), P = 0.079; men HR (95% CI) 1.17 (0.93–1.46) P = 0.159; Postmenopausal women HR (95% CI) 1.12 (0.93–1.46) P = 0.170]. A similar lack of association was observed when the analysis was restricted to subjects without high levels of fibrinogen and/or GGT [HRs (95% CI) pre-menopausal women 1.38 (0.95–1.99), P = 0.085; men 1.11 (0.92–1.34), P = 0.246); post-menopausal women 0.92 (0.66–1.29), P = 0.658]. There were no statistically significant associations between serum ferritin, as Z score or quantiles (lowest vs. highest), and CEVD or CHD in the analyses by sex and menopausal status. Combining data for pre- and post- menopausal women in a single category and adjusting for menopausal status did not result in significant associations across the above mentioned sensitivity analyses.

### Multivariate models

Additionally, we verified the associations between the covariates used for adjustments and CMD to create multivariate models with variables significantly associated. All the covariates used in the full adjustment (Table 2) were significantly associated with development of type 2 diabetes in a univariate analysis (P value < 0.1 as filter to enter in the multiple analysis). In the multiple analyses for type 2 diabetes the variables in the final a model after backwardly removing variables with P value > 0.05 were age, GGT levels, smoking, alcohol consumption, BMI, HDL-C levels and diastolic blood pressure. For CHD final model was composed by age, sex/menopausal status, smoking, HDL-C levels, total cholesterol levels and diastolic blood pressure. See Models in Additional file 1: Table 3. When used the above multivariate models to adjust the associations between ferritin and incident CMD, the associations described in Table 2 practically remained unaltered.

### Use of medications or vitamins/supplements, and comorbidities across ferritin levels

Additional file 1: Table S4 describes use of medications and vitamin or dietary supplements in the total sample and by quartiles of ferritin levels. Proportions for use medications were low given that the sample was CMD free at baseline of the study. Only use of anti-hypertensive drugs showed difference across ferritin levels being slightly higher in the highest quartile. When adding the use of each kind of medication/supplement as covariates in the full adjusted models did not substantially affect the associations between ferritin levels and each CMD previously described in Table 2 and Fig. 1.

Regarding comorbidities, Additional file 1: Table S5 describes proportions of individuals with several types of long-standing comorbidities at baseline of the study across quartiles of serum ferritin. Only having diseases affecting musculoskeletal system (e.g. osteoarthritis, rheumatoid arthritis, sarcopenia, hip dysplasia, etc.) significantly varied across ferritin levels with a higher proportion of these cases in higher ferritin levels. However, further adjustment for these comorbidities did not substantially affect the associations between ferritin levels and incident CMD.

Other analyses related to the adjustment for C reactive protein (SHeS 1998) and exclusion of individuals according to high levels of ferritin, fibrinogen and GGT levels are reported in the Additional file 1.

## Discussion and conclusions

Excess iron, and specifically free iron, is known to trigger the production of reactive oxygen species. Iron and inflammation are intertwined in a bidirectional relationship. Iron potentiates the inflammatory phenotype and inflammatory cells secrete inflammatory mediators such as cytokines and nitric oxide, implicated in the pathophysiology of T2D and CMD. In the present study, we have reported the associations between iron stores and incidence of several CMDs in a nationally representative population at high cardiovascular risk over a mean follow-up of 14 years. We found a statistically significant association between high ferritin and development of CEVD when we used higher percentiles to define high ferritin concentration. Interestingly, these cut-off points were still within the normal reference values of ferritin. The findings also confirm previous observations of an association between serum ferritin and T2D and no evidence of an association with CHD.

### Ferritin and T2D

The results for the association between serum ferritin and incident T2D are consistent with the positive and significant association reported in a meta-analysis of prospective studies published up to 2012 [1]. In this meta-analysis, the pooled relative risk (95% CI) for incident T2D for the highest ferritin quintile vs. the lowest quintile was 1.73 [1]. The association in the meta-analysis is slightly stronger than that in the Scottish population studied here [HR (95% CI) 1.59 (1.10–2.34), P = 0.006], presumably because several studies of the meta-analysis lacked adjustment for transaminase levels. In the present study, the HR for the association between highest compared to lowest quartile of ferritin levels and incident T2D decreased by 16% when GGT levels were added to the adjustment model. Some few studies failed to find a significant independent association between serum ferritin and development of T2D. A case-cohort study of the Atherosclerosis Risk in Communities study (ARIC) (599 cases and 690 controls) did not find a significant association between ferritin levels in the highest quintile (v. the lowest) and T2D after adjustment for BMI and traditional cardiovascular risk factors [HR 95% CI 0.81 (0.49–1.34) [21]. However, the study did not disclose which covariate/s had more weight in the attenuation of the association. In a recent age and sex-matched case–control prospective study (327 cases, 641 controls) involving Japanese workers, the association between ferritin and incident T2D was weakened after further adjustment for transaminase and lipid levels [HR (95% CI) 1.40 (1.01–1.93), P = 0.02 before adjustment; HR (95% CI) 1.20 (0.86–1.67), P = 0.16 after adjustment] [22]. It is possible that limited statistical power explains the non-statistically significant findings in the ARIC and Japanese cohorts. A large study conducted in a sub-cohort of the European Multicenter InterAct study found a significant association between serum ferritin and T2D in both sexes [23]. The association was markedly attenuated in men but not in women when individuals with signs of inflammatory or hepatic disease, high alcohol intake, and who were overweight were excluded from the analysis. Similarly, the association was stronger for women but not for men when analyses were restricted to ferritin values lower than 1000 µg/L. However, the associations in women were no longer observed when standardized units of ferritin were used. Our findings are consistent with these observations Apparently stronger associations with T2D in women using natural units of ferritin in some analyses might be a statistical artifact arising from the different distributions of ferritin in men and women, as suggested in the InterAct study [23]

### Ferritin and CEVD

As previously mentioned, there have been very few studies on ferritin and CEVD in general populations, and those that exist show inconclusive findings. Two longitudinal studies involving 1,134 Dutch post-menopausal women (aged 40–70 years, mean follow-up, 4.3 years) [24] and a sub-cohort (n = 1612) of the Busselton Health study (age 40–89 years,17-years of follow-up) [25] reported similar positive associations between serum ferritin levels in the highest tertile (vs. lowest) and stroke of any subtype but without reaching statistical significance in fully adjusted models [HR (95%CI) 1.45 (0.76–3.85) and 1.43 (0.78–2.64), respectively]. Analyses by sex in the Busselton Health study did not show statistically significant associations either. Both studies had comparable age ranges and number of incident cases of stroke (Dutch cohort, 63 cases; Busselton Health study’s sub-cohort, 55 women and 63 men). In the Dutch cohort of post-menopausal women, by using serum ferritin ≥ 200 µg/L compared to ferritin lower than that cut-off point, the positive association between ferritin and stroke of any subtype was borderline statistically significant [HR (95%CI) 1.77 (1.03–3.05)]. Although the cut-off point for highest tertile of ferritin for men in the Busselton Health study was 233 µg/L, for women the cut-off point for this tertile was only 122 µg/L. Therefore, it is uncertain whether by comparing extreme values of ferritin, the association with stroke might have been strengthened in the Busselton Health Study.

By exploring additional higher cut-off points for increased ferritin, the association between serum ferritin and CEVD became more evident. Since CEVD is less common than T2D and CHD, it is likely that clearer associations can be observed when incident and/or prevalent cases are more concentrated into very high categories of distribution of a predictor variable, such as iron stores. Despite using sextiles of ferritin distribution, the cut-off points defining increased ferritin were still within the normal range for ferritin levels, and the exclusion of subjects with ferritin values > 300 µg/L did not affect the ferritin-CEVD association. This suggests that there may be an increased risk of CEVD in the general population that does not have extremely high concentrations of stored iron if this is not a chance finding. However, a graphical evaluation of the ferritin-CEVD relationship by using standardised values of ferritin in our analysis suggested, a threshold effect. Studies with more incident CEVD cases are required to establish whether this is a true finding, since statistical tools for testing non-linearity demand high statistical power. Our analysis, to the best of our knowledge, is the first attempt to graphically describe the shape of the association between serum ferritin level and the risk of CEVD.

### Ferritin and the lack of an independent association with CHD

Serum ferritin levels were associated with CHD in unadjusted and age/sex-menopausal status-adjusted models but not in the fully-adjusted model used in these analyses. In this latter model, several cardiovascular risk factors, such as cholesterol markers, SBP, and BMI, markedly attenuated the ferritin-CHD association (data not shown). The lack of an independent association is consistent with the findings from a recent meta-analysis by Das et al. on several iron markers and CHD which evaluated 17 prospective studies with a total of 9,236 cases of CHD and 156,427 participants [2]. In this meta-analysis, the pooled association between serum ferritin in the highest tertile (vs. lowest tertile) and CHD was not significant [OR 95% (CI) 1.03 (0.87–1.23)]. Paradoxically, transferrin saturation was significantly and inversely associated with incident CHD. The authors acknowledged difficulties in inferring causality due to potential reverse causality and residual confounding. However, they did not ignore the likely role of anaemia in the inverse association, since a higher iron status could prevent the onset of anaemia, which is associated with symptomatic CHD[2]. It is still unclear why the directions of associations with iron status markers are inconsistent. If iron deficiency and anaemia are related to CHD, the effect estimates for the relationship with serum ferritin should show an inverse significant pattern as well. This discrepancy reinforces the notion that the iron markers are differentially associated with cardiometabolic risk through mechanisms other than iron metabolism.

The fully adjusted effect estimate described in this work for the non-significant positive association between high ferritin (comparison of extreme quartiles), [HR (95%CI) 1.08 (0.76–1.52)] is similar to those reported in three studies out of ten from the meta-analysis by Das et al. on ferritin and CHD [2]. Among the remaining seven studies, four reported effect estimates much lower than 1.0, and three, higher than 1.0. However, Das et al. did not find any source of statistically significant heterogeneity among factors of location, degree of adjustment for confounders, sex, or case definition across meta-regression analyses [2].

### Limitations and strengths

Several limitations need to be acknowledged. SHeS did not measure serum triglycerides, and it is unknown to what extent this risk factor could have attenuated the associations found. Additionally, blood samples in the SHeS were not taken at fasting state, and although none of the biomarkers measured required fasting is important to observe differences or similarities with future studies using fasted samples. Two issues may have led to underestimating the total number of cases of T2D and people with undiagnosed diabetes may have been included in the non-diabetic population. First, there could be an underestimation of incident T2D cases between 1995 and 2003, because cases of T2D for people who died before 2004 were only identified using information on hospital admissions, since the Scottish Diabetes Register is only thought to have been complete since 2004. There is also potential for erroneous inclusion of people with undiagnosed diabetes both at baseline and follow-up in the non-diabetic group. However, the above issues did not affect the statistical power to find a consistent association between ferritin and T2D in the Scottish population, and this may be related to the long follow-up of this study, which is the longest to date. Regarding cardiovascular disease, we have not identified CHD or CEVD without hospital admission.

Future studies on iron and cardiometabolic disease should explore additional iron markers since SHeS, the source of this investigation, only had available serum ferritin. However, it is important to take into account that serum ferritin is the iron marker most consistently associated with insulin resistance and metabolic syndrome. Markers such as soluble transferrin receptors or transferrin have shown inconclusive findings among studies and opposite associations with cardiometabolic risk to those found for serum ferritin. This latter observation might reflect different pleiotropic influences and/or roles, besides iron metabolism, for each iron marker. Therefore, is highly relevant to characterize the relationship of several iron markers and risk of different cardiometabolic diseases in large samples and prospective studies as this study has done with serum ferritin.

Despite the longitudinal significant associations between serum ferritin and CMD described in this study, we cannot establish that there is causal relationship. In terms of the adjustments use in our analysis, it seems that inflammation, liver injury and well-known cardiometabolic risk factors would not influence or confound the ferritin-CMD association. However, residual confounding may persist given the high chance of ferritin might behave in relation to cardiometabolic risk on the basis of biological roles o pleiotropic influences others than iron metabolism. Therefore, future studies should try to test new potential co-variables or confounding factors related to different kinds of metabolic and endocrine pathways and biological events (e.g. thyroid function markers, oxidative stress). In this same way, the role of nutraceuticals should be specifically addressed in upcoming research since these food ingredients might affect ferritin levels and /or cardiometabolic risk [26]. In the present study, we did not find effects of use of vitamin and dietary supplements on the ferritin-CMD associations. However, there was not available precise information on the specific nutraceuticals used by the subjects.

The proportion of excluded cases with missing values for exposure/outcome variables or covariates may have introduced bias in the associations found. Estimation of outcome variables might have been affected by the loss of follow-up. If the outcomes were overestimated, the associations with serum ferritin may have been biased toward the null hypothesis, as in the case of CHD. If the outcomes had been underestimated, it is possible that the loss of statistical power led to weaker associations, as observed for CEVD. Time trends in background interventions for prevention and treatment of cardiovascular disease may mean that it is inappropriate to extrapolate findings from the SHeS analysis to contemporary populations given the long follow-up period for SHeS participants. For instance, the proportions of people who take aspirin and statins have increased over that time period and these treatments have been linked (along with angiotensin converting enzyme inhibitors, thrombolysis, and coronary artery bypass graft surgery) to a 25–55% of the fall in cardiovascular mortality rates in Scotland (2000–2010) [27]. It is not clear how this might have affected the association between ferritin and cardiovascular outcomes.

To the best of our knowledge, this study is the first to have simultaneously evaluated the association between ferritin and incidence of several CMDs. Moreover, this is the first study in a nationally representative population. The study also explored different upper categories of ferritin concentration in relation to the risk of CMD and evaluated the potential non-linear relationships between ferritin and each CMD outcome. Our findings along with previous literature remark the importance of a deeper evaluation of high ferritin levels in daily clinical practice since iron deficiency uses to be the main concern. An emerging body of evidence has linked serum ferritin levels to major clinical outcomes such as cardiovascular mortality in patients with type 2 diabetes. This has the potential to attribute to serum ferritin the value of risk stratification and prognosis, regardless of causal or non-causal relationship. Future efforts are required to fully explore this potential in well-designed prospective large-scale cohorts.

In conclusion, serum ferritin levels were positively and independently associated with incident T2D, and with incident CEVD if higher cut-off points for upper ferritin levels were considered. The lack of an independent association between serum ferritin and CHD reported in previous studies was confirmed in this Scottish population. Further studies on ferritin and CEVD are required to confirm the association described here.

## Supplementary Information

**Additional file 1:** **Figure S1.** Identification of the cohort SHeS 95-98. Among the individuals removed from the sample for having no linkage to Scottish Morbidity Record (SMR): 14 (0.3%) had dead status, 323 (7.8%) migrated outwith Scotland, 1603 (38.6%) did not consent to data linkage, 264 (6.4%) had not linkage to Community Health Index (CHI) , and 1952 (47%) had CHI but had not reason described for not linkage to SMR. **Figure S2.** Adjusted hazard ratios for CEVD by sex/menopausal specific Z score of ferritin levels. **Table S1.** HRs and 95% CI for the incidence of diabetes and cardiovascular diseases by serum ferritin quintiles with middle quintile as reference. **Table S2.** HRs and 95% CI* for the incidence of diabetes and cardiovascular diseases by serum ferritin levels (weighted analysis). **Table S3.** Final multivariate models for each cardiometabolic disease. **Table S4.** Use of medicines and vitamin/dietary supplements at baseline by sex-and menopausal status-specific quartiles of ferritin level in the study cohort. **Table S5.** Types of comorbidities in by ferritin levels in the individuals of the study

## Data Availability

The datasets used and/or analysed during the current study are available from the corresponding author on reasonable request.

## Abbreviations

CMD: Cardiometabolic disease
T2D: Type 2 diabetes
CHD: Coronary heart disease
CEVD: Cerebrovascular disease
SHeS: Scottish health survey
T1D: Type 1 diabetes
GGT: Gamma-Glutaryltransferase
BMI: Body mass index
SBP: Systolic blood pressure
DBP: Diastolic blood pressure
WC: Waist circumference
CRP: C reactive protein
