# Supplementary material for: Serum ferritin and incident cardiometabolic diseases in Scottish adults
Source: Cardiovasc Diabetol. 2022 Feb 16;21:26. doi: 10.1186/s12933-022-01450-7 (PMC8851777; doi:10.1186/s12933-022-01450-7)
Supplement: Supplementary file 1 — Additional file 1: Figure S1. Identification of the cohort SHeS 95-98. Among the individuals removed from the sample for having no linkage to Scottish Morbidity Record (SMR): 14 (0.3%) had dead status, 323 (7.8%) migrated outwith Scotland, 1603 (38.6%) did not consent to data linkage, 264 (6.4%) had not linkage to Community Health Index (CHI) , and 1952 (47%) had CHI but had not reason described for not linkage to SMR. Figure S2. Adjusted hazard ratios for CEVD by sex/menopausal specific Z score of ferritin levels. Table S1. HRs and 95% CI for the incidence of diabetes and cardiovascular diseases by serum ferritin quintiles with middle quintile as reference. Table S2. HRs and 95% CI* for the incidence of diabetes and cardiovascular diseases by serum ferritin levels (weighted analysis). Table S3. Final multivariate models for each cardiometabolic disease. Table S4. Use of medicines and vitamin/dietary supplements at baseline by sex-and menopausal status-specific quartiles of ferritin level in the study cohort. Table S5. Types of comorbidities in by ferritin levels in the individuals of the study [file 12933_2022_1450_MOESM1_ESM.docx]

**figure S1**


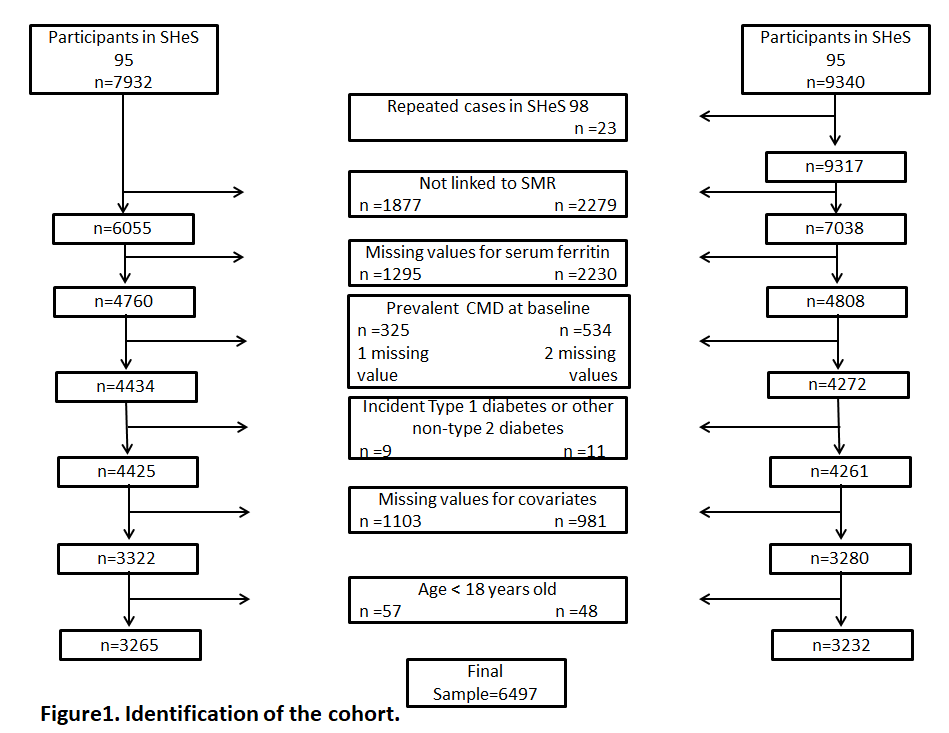


### figure S1. Identification of the cohort SHeS 95-98. *Among the individuals removed from the sample for having no linkage to Scottish Morbidity Record (SMR): 14(0.3%) had dead status, 323 (7.8%) migrated outwith Scotland, 1603 (38.6%) did not consent to data linkage, 264(6.4%) had not linkage to Community Health Index (CHI) , and 1952 (47%) had CHI but had not reason described for not linkage to SMR.*

figure S2. Adjusted hazard ratios for CEVD by sex/menopausal specific Z score of ferritin levels.

**
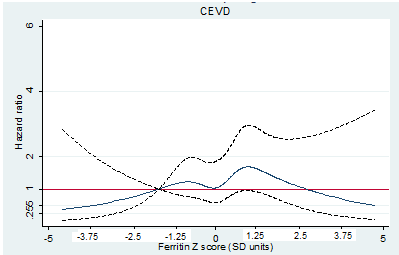
**

| **Table S1. HRs and 95% CI for the incidence of diabetes and cardiovascular diseases by serum ferritin quintiles with middle quintile as reference** | | | |
| --- | --- | --- | --- |
|  | **Type 2 diabetes** | **Coronary heart disease** | **Cerebrovascular disease** |
| **Ferritin** |  |  |  |
| **Quintile 1** | 0.87  (0.54-1.41) | 1.25  (0.85-1.85) | 0.93  (0.52-1.68) |
| **Quintile 2** | 1.51  (0.99-2.31) | 0.75  (0.49-1.14) | 1.28  (0.71-2.28) |
| **Quintile 3** | 1.00 (reference) | 1.00 (reference) | 1.00 (reference) |
| **Quintile 4** | 1.47  (0.98-2.20) | 1.03  (0.71-1.49) | 1.58  (0.90-2.75) |
| **Quintile 5** | **1.82**  **(1.25-2.65)*** | 1.07  (0.73-1.56) | 1.45  (0.81-2.57) |

*** Adjusted for age, sex/menopausal status, fibrinogen levels, GGT levels, alcohol intake, smoking, systolic blood pressure, diastolic blood pressure, total cholesterol, HDL cholesterol, body mass index and year of survey.**

****P <0.05***

| **Table S2**. **HRs and 95% CI* for the incidence of diabetes and cardiovascular diseases by serum ferritin levels (weighted analysis)** | | | | | | | | | |
| --- | --- | --- | --- | --- | --- | --- | --- | --- | --- |
|  | Type 2 diabetes | | | CHD | | | CEVD | | |
|  | Premenopausal women | Postmenopausal women | Men | Premenopausal women | Postmenopausal women | Men | Premenopausal women | Postmenopausal women | Men |
| Z score of log-ferritin | 1.25  (0.92-1.69)  P=0.149 | 1.14  (0.87-1.48)  P=0.325 | **1.20**  **(1.01-1.43)**  **P=0.033** | 1.01  (0.70-1.46)  P=0.9239 | 1.00  (0.80-1.26)  P=0.940 | 1.01  (0.87-1.18)  P=0.818 | 1.32  (0.88-1.98)  P=0.169 | 1.04  (0.76-1.41)  P=0.799 | 1.11  (0.88-1.39)  P=0.357 |
| *Ferritin* |  |  |  |  |  |  |  |  |  |
| Quartile 1 | 1.00 (reference) | 1.00 (reference) | 1.00  (reference) | 1.00 (reference) | 1.00 (reference) | 1.00  (reference) | 1.00 (reference) | 1.00 (reference) | 1.00  (reference) |
| Quartile 2 | 1.76  (0.50-6.14) | 0.85  (0.46-1.57) | 0.86  (0.47-1.56) | 1.48  (0.48-4.58) | 1.08  (0.55-2.13) | 0.76  (0.48-1.22) | 3.4  (1.05-11.5) | 0.57  (0.25-1.28) | 1.05  (0.49-2.24) |
| Quartile 3 | 2.43  (0.77-7.69) | 0.79  (0.40-1.56) | 0.89  (0.50-1.60) | 0.73  (0.23-2.31) | 0.85  (0.43-1.69) | 0.74  (0.48-1.16) | 1.52  (0.38-6.02)) | 0.91  (0.43-1.93) | 1.02  (0.46-2.25) |
| Quartile 4 | 2.26  (0.67-7.61) | 1.27  (0.66-2.42) | 1.47  (0.87-2.48) | 0.83  (0.28-2.48) | 1.34  (0.68-2.62) | 1.00  (0.65-1.53) | 3.37  (0.97-11.7) | 0.75  (0.32-1.73) | 1.55  (0.72-3.31) |
| * Adjusted for age, sex/menopausal status, fibrinogen levels, GGT levels, alcohol intake, smoking, systolic blood pressure, diastolic blood pressure, total cholesterol, HDL cholesterol, body mass index and year of survey | | | | | | | | | |

| **Table S3. Final multivariate models for each cardiometabolic disease.** | | | | | |
| --- | --- | --- | --- | --- | --- |
| **T2D** | | CHD | | CEVD | |
| Variable | HR (95% IC) | Variable | HR (95% IC) | Variable | HR (95% IC) |
| Age (years) | 1.05  (1.04-1.06)  P<0.001 | Age (years) | 1.06  (1.05-1.07)  P<0.001 | Age (years) | 1.02  (0.90-1.15)  P=0.669 |
| GGT (UI/mL) | 1.005  (1.003-1.006) | Sex/menopausal status |  | Smoking |  |
| Smoking |  | *Premenopausal women* | 1.00  (reference) | *Never smoker* | 1.00  (reference) |
| *Never smoker* | 1.00  (reference) | *Postmenopausal women* | 1.17  (0.70-1.94)  P=0.541 | *Ex-regular or Ex-occasional smoker* | 0.74  (0.47-1.17)  P=0.210) |
| *Ex-regular or Ex-occasional smoker* | 1.01  (0.75-1.37)  P=0.917 | *Men* | 1.96  (1.27-3.01)  P=0.002 | *Current smoker* | 1.74  (1.19-2.54)  P=0.004 |
| *Current smoker* | 1.47  (1.08-2.00)  P=0.013 | Smoking |  | Diastolic pressure mmHg | 1.01  (1.009-1.02)  P<0.001 |
| Alcohol consumption |  | *Never smoker* | 1.00  (reference) |  |  |
| *Never drank* | 1.00  (reference) | *Ex-regular or Ex-occasional smoker* | 1.25  (0.90-1.75)  P=0.180 |  |  |
| *Ex-drinker* | 0.65  (0.30-1.38)  P=0.267 | *Current smoker* | 2.34  (1.74-3.14)  P<0.001 |  |  |
| *Trivial drinker/Non-zero but under 1* | 0.63  (0.36-1.10)  P=0.528 | Total cholesterol mmol/L | 1.16  (1.05-1.29)  P=0.004 |  |  |
| *1-20* | 0.85  (0.53-1.37)  P=0.528 | HDL-C mmol/L | 0.48  (0.35-0.68)  P<0.001 |  |  |
| *≥ 21* | 0.57  (0.32-0.99)  P=0.049 | Systolic pressure mmHg | 1.16  (1.05-1.29)  P=0.004 |  |  |
| BMI (Kg/mts^2^) | 1.15  (1.13-1.18)  P<0.001 |  |  |  |  |
| HDL-C mmol/L | 0.37  (0.25-0.56)  P<0.001 |  |  |  |  |
| Diastolic pressure mmHg | 1.008  (1.001-1.01) P=0.011 |  |  |  |  |
|  |  |  |  |  |  |

| **Table S4. Use of medicines and vitamin/dietary supplements at baseline by sex-and menopausal status-specific quartiles of ferritin level in the study cohort** | | | | | | |
| --- | --- | --- | --- | --- | --- | --- |
|  | **All**  **n=6497** | **Q1**  **n=1615** | **Q2**  **n=1611** | **Q3**  **n=1645** | **Q4**  **n=1626** | **P for trend** |
| **Anti-hypertensive** | 427  (9.2 %)  Missing =1867 | 78  (7.4 %)  Missing =561 | 108  (9.2%)  Missing  =439 | 97  (8.1%)  Missing  =453 | 144  (11.9 %)  Missing  =414 | **0.001** |
| **Lipid-lowering** | 7  (0.2%)  Missing =1878 | 0  (0%)  Missing =561 | 2  (0.2%)  Missing =443 | 1  (0.1%)  Missing =455 | 4  (0.2%)  Missing =419 | 0.149 |
| **Iron supplement** | 70  (1.5 %)  Missing =1860 | 14  (1.3 %)  Missing =554 | 20  (1.7%)  Missing =440 | 16  (1.3%)  Missing =452 | 20  (1.2%)  Missing =414 | 0.079 |
| **Vitamin or dietary supplements** | 1574  (24.2 %)  Missing=5 | 384  (23.8%)  Missing=1 | 403  (25 %)  Missing =2 | 401  (24.4 %)  Missing =0 | 386  (23.7 %)  Missing =0 | 0.876 |
| **Hormonal contraception (oral, injections, intrauterine device) *** | 272  (15.2 %)  Missing  =1795 | 76  (15.3)  Missing=381 | 70  (15.3%)  Missing =433 | 61  (14.8 %)  Missing =505 | 65  (15.5 %)  Missing =476 | 0.968 |
| **Antiplatelet (only available in SheS 1995)** | 19  (0.6 %)  Missing =3279 | 4  (0.6 %)  Missing =909 | 10  (1.2%)  Missing =808 | 2  (0.2%)  Missing =808 | 3  (0.4%)  Missing =786 | 0.149 |
| *Missing values are on the basis of female gender | | | | | | |

| **Table S5. Types of comorbidities in by ferritin levels in the individuals of the study** | | | | | | |
| --- | --- | --- | --- | --- | --- | --- |
|  | **All**  **n=4415*** | **Q1**  **n=1148*** | **Q2**  **n=1084*** | **Q3**  **n=1088*** | **Q4**  **n=1095*** | **P for trend** |
| Neoplasms | 71(1.6%) | 16(1.4%) | 18(1.7%) | 18(1.7%) | 19(1.7%) | 0.541 |
| Endocrine & metabolic | 130(2.9%) | 30(2.6%) | 39(3.6%) | 23(2.1%) | 38(3.5%) | 0.619 |
| Mental disorders | 195(4.4%) | 56(4.9%) | 47(4.3%) | 48(4.4%) | 44(4%) | 0.359 |
| Nervous System | 220(5%) | 62(5.4%) | 50(4.6%) | 53(4.9%) | 55(5%) | 0.752 |
| Eye problems | 91(2.1%) | 24(2.1%) | 22(2%) | 23(2.1%) | 22(2%) | 0.032 |
| Ear problems | 95(2.2%) | 17(1.5%) | 24(2.2%) | 31(2.8%) | 23(2.1%) | 0.192 |
| Respiratory system | 511(11.6%) | 124(10.8%) | 124(11.4%) | 134(12.3) | 129(11.8%) | 0.369 |
| Digestive system | 329(7.5%) | 90(7.8%) | 81(7.5%) | 83(7.6%) | 75(6.8%) | 0.423 |
| Genito-urinary system | 130(2.9%) | 29(2.5%) | 41(3.8%) | 24(2.2%) | 36(3.3%) | 0.735 |
| Skin problems | 124(2.8%) | 31(2.7%) | 30(2.8%) | 37(3.4%) | 26(2.4%) | 0.883 |
| Musculoskeletal system | 1009(22.9%) | 239(20.8%) | 236(21.8%) | 249(22.9%) | 285(26%) | **0.003** |
| Infectious Disease | 13(0.3%) | 3(0.3%) | 3(0.3%) | 4(0.4%) | 3(0.3%) | 0.857 |
| Blood & related organs | 31(0.7%) | 6(0.5%) | 10(0.9%) | 9(0.8%) | 6(0.5%) | 0.415 |
| *Sample size is smaller than the sample used in the main analysis because of missing values for comorbidities. | | | | | | |

**Additional sub-analyses and sensitivity analyses**

The exclusion of individuals with serum ferritin >300 µg/L or further adjustment for self-reported hypertension or physical activity and WC did not substantially affect the associations previously described. The ferritin-CEVD association remained unaffected when cases of subarachnoid haemorrhage were excluded (n=16). In participants from SHeS 1998, further adjustment for CRP levels did not alter the significant association between serum ferritin in the highest quartile (vs. lowest quartile) and incident T2D [unadjusted HR (95% CI): 2.18(1.22–3.90), P = 0.008; fully adjusted HR (95% CI): 2.16(1.20–3.89), P = 0.010]. In this same sub-cohort, ferritin and incident CHD and CEVD were not significantly associated, and additional adjustment for CRP did not modify these non-significant associations.

When the analysis was restricted to individuals without signs of inflammation and/or hepatic injury (n=5626), the association between ferritin, as upper quartile or continuous variable, and T2D was attenuated [ferritin Z score HR (95% CI) 1.12 (0.97–1.31), P = 0.105; higher quartile vs. lowest HR (95% CI) 1.32 (0.87–2.09), P = 0.177]. However, by comparing extreme quintiles, the association was statistically significant [HR (95% CI) 1.67 (1.02–2.75), P = 0.041]. The association between ferritin (extreme sextiles compared) with CEVD remained similar [HR (95% CI) 2.03 (1.01–4.08) P = 0.045].
